# Asymptomatic *Plasmodium falciparum* parasitaemia among pregnant women: a health facility based survey in Nassarawa-Eggon, Nigeria

**Published:** 2017-06-01

**Authors:** Samuel E. Emiasegen, Fatima J. Giwa, Olufemi Ajumobi, IkeOluwapo O. Ajayi, Saad A. Ahmed, Adebola T. Olayinka

**Affiliations:** 1 Nigeria Field Epidemiology and Laboratory Training Programme, Abuja, Nigeria; 2 Department of Medical Microbiology, Ahmadu Bello University, Zaria, Nigeria; 3 Surveillance and Data Management Unit, National Malaria Elimination Programme, Federal Ministry of Health, Abuja, Nigeria; 4 Department of Epidemiology and Medical Statistics, Faculty of Public Health, University of Ibadan, Nigeria; 5 Pathology Department, Ahmadu Bello University, Zaria, Nigeria

## Abstract

**Background:**

Asymptomatic malaria parasitaemia remains an effective transmission pool for malaria during pregnancy, which can result in placenta parasitaemia and adverse pregnancy outcomes. This study examined asymptomatic malaria parasitaemia among pregnant women in the antenatal clinic in General Hospital, Nassarawa-Eggon, Nasarawa State, Nigeria.

**Materials and methods:**

A cross-sectional hospital based survey was carried out among 242 apparently healthy pregnant women presenting for booking in an antenatal clinic between June and August 2014. An interviewer-administered semi-structured questionnaire was used to obtain information on socio-demographic data and possible risk factors for asymptomatic malaria parasitaemia. These women should not have taken antimalarial medicines two weeks prior to the interview. Microscopy was used to identify malaria parasites and haemoglobin levels were estimated. Data was analysed using Epi Info 3.5.3. Descriptive statistics such as means, standard deviations, proportions, and range were used to summarise the data and the Chi square test was used to test association between categorical explanatory variables and outcome variables.

**Results:**

Mean age (± SD) was 25.5 ± 5.5 years, 118 (48.8%) of the women were in the 25–34 years age group, while 153 (63.2%) were multigravidae. Asymptomatic *Plasmodium falciparum* infection was found in 55 women (22.7%; 95% CI: 18.0-28.7%) Among these, 36 (65.5%) were anaemic [OR: 2.0, CI: 1.1-3.8]. Long lasting insecticidal net (LLIN) was not used by 17 (30.9%) of the respondents. Younger age group (below 25 years) [AOR: 2.4, CI: 1.2-4.9] and non-usage of LLIN [AOR: 2.4, CI: 1.1-5.1] were significant predictors of asymptomatic malaria parasitaemia.

**Conclusion:**

Asymptomatic malaria parasitaemia is a health challenge among pregnant women, especially in the younger age group and can predispose them to maternal anaemia. The supply and appropriate use of LLIN should be intensified.

## 1 Introduction

Every year, globally some 125 million pregnant women and approximately half of the world’s population are at risk of contracting malaria [1,2]. Most cases and deaths occur in sub-Saharan Africa [3]. In Nigeria, 97% of the population is at risk of malaria with 11% maternal deaths attributable to this disease [4]. The frequency and severity of malaria are greater in pregnant than in the non-pregnant women and malaria in pregnancy causes serious adverse effects including abortion, low birth weight, and maternal anaemia [5].

Malaria during pregnancy has been reported to occur more in younger women, primigravidae and secundigravidae [6]. Age and pregnancy-associated anti-parasite immunity have been identified to play important roles in *P. falciparum* infection. A study in Asia identified age as an independent risk factor for fatal outcomes in malaria infection [7]. The severity of malaria parasitaemia in pregnancy was also reported to be higher in younger pregnant women from studies undertaken in Nigeria and Cameroon [8,9].

In Nigeria, *P. falciparum* is the most endemic species and the most fatal among pregnant women, children and the immunocompromised [10]. Most malaria infections among pregnant women in areas of high or moderate transmission are asymptomatic and infected women may not seek treatment with a consequent outcome of maternal anaemia and severe malaria during pregnancy [11]. The clinical consequences of asymptomatic malaria parasitaemia are not fully understood and it is generally assumed that in endemic areas asymptomatic parasitaemia results in the development of partial immunity [12].

Asymptomatic malaria parasitaemia has been identified to provide a reservoir for malaria transmission as well as a precursor in the progression to symptomatic disease [12]. However, women who live in areas of stable malaria transmission have been identified with greater immunity and experiencing fewer symptoms during episodes of malaria but they commonly develop severe anaemia as a consequence of the infection [13], which are often asymptomatic.

National Malaria Control and Elimination Programmes are geared towards the protection of pregnant women living in malaria-endemic zones because of their reduced immunity [14]. Reports by Schantz-Dunn and Nour identified that malaria can directly contribute to almost 25% of maternal deaths each year [10]. Despite these implications, little attention is given to asymptomatic malaria parasitaemia in Nigeria. We conducted this study to examine the prevalence and possible factors associated with asymptomatic malaria parasitaemia in pregnant women to provide evidence-based information to plan for effective prevention, control, and elimination of malaria among pregnant women in Nasarawa state and the nation at large.

## 2 Materials and methods

### 2.1 Study location

Nassarawa-Eggon Local Government Area (LGA) is centrally located in the State, and consists of both urban and rural populace. The climate of the LGA falls within the tropical sub-humid climate with two clearly marked rainy (May to October) and dry (November to April) seasons, but also experience the northeast trade winds and thus the dry harmattan [15]. HIV prevalence in Nassarawa-Eggon LGA is 2-4% and malaria transmission is meso-endemic and seasonal.

This study was conducted at the General Hospital, Nassarawa-Eggon, the only secondary and referral health facility in Nassarawa-Eggon LGA of Nasarawa state, north central Nigeria. The hospital has a maternity ward for pregnant women and 31 beds. The facility carries out antenatal clinic (ANC) activities twice-weekly. The clinics are manned by qualified nurses and midwives and routine care, intermittent preventive therapy (IPT) and relevant investigations are provided to attending pregnant women. Besides the mass bednet distribution campaign in 2014 by the National Malaria Elimination Programme (NMEP) supported by the Malaria Action Programme for states (MAPs) in the area, long lasting insecticide nets (LLINs) are also distributed to pregnant women attending ANC routinely.

### 2.2 Study design

A cross-sectional study was conducted from June to August, 2014. Healthy pregnant women (with no history of fever, no fever, and no symptoms suggestive of malaria) attending ANC for the first time in their current pregnancy, who had not been treated with antimalarial medicine in the preceding two weeks, were recruited at the hospital. All women with a body temperature > 37.5oC were excluded. Asymptomatic malaria parasitaemia was defined as the presence of asexual parasites in the blood without symptoms of illness and temperature of < 37.5oC.

The sample size was calculated using a prevalence of 14% from a study on the prevalence of maternal peripheral malaria parasitaemia at delivery in Minna, north central Nigeria in 2012 [16]. A 5% precision and 95% confidence level was used for the estimation. A sample size of 185 was therefore calculated and with the consideration of a non-response rate of 10% a sample size of 206 was estimated and made up to 242 for the study.

### 2.3 Data collection

A semi-structured pretested interviewer-administered questionnaire was used to obtain information on socio-demographics and possible factors associated with asymptomatic malaria parasitaemia. These factors include age, occupation, educational level, regular use of an LLIN, gestational age, and gravidity. Since recruitment was at the first visit, questions on IPT were not asked but the women were given IPT after the interview. The questionnaire was developed in English language using questions adopted from literature on related studies and also questions based on knowledge of the subjects by the investigators [8,14]. The questionnaire was translated to the local language by trained interviewers who were local nurses. It was back-translated to English by a local medical doctor to ensure correct translation. The interviews were conducted by trained interviewers, after the pregnant women had been attended to, in a secluded room, in the clinic.

### 2.4 Laboratory methods

About 2-3 ml of peripheral venous blood was aseptically collected from each participant into EDTA tubes by a trained laboratory technician. Thick and thin blood films were prepared on glass slides for parasite identification and speciation using Giemsa technique [17]. The slides were stained and viewed using x100 oil immersion objective lens. At least 100 high power fields were examined before a thick smear was reported as negative. Each slide was read independently by two trained microscopists and slides were reported as positive when both microscopists agreed on the reading. A third trained microscopist was employed to read slides with discrepancies. Blood film slides without parasites identified were reported as No Malaria Parasites Seen [NMPS]. Capillary tubes were used to obtain blood from the samples and haemoglobin levels were estimated using the haematocrit technique [18]. Anaemia was defined as a haemoglobin level <11.0 g/dl or packed cell volume <33.0% [19].

### 2.5 Data analysis

Completed questionnaires and laboratory results were reviewed prior to data entry to exclude incomplete and inaccurate data. Epi Info software version 3.5.3 was used for both data entry and data analysis [20]. The dependent variable was presence of parasitaemia and the independent variables were socio-demographic characteristics and likely associated factors (such as age, educational level, occupation, use of LLIN, and gravidity). Gestational age was determined by the attending midwife or doctor. For the purpose of this study, regular use of LLIN was defined as sleeping inside an LLIN every night (daily at least for the last 3 nights before the interview), not just the night before. Univariate and bivariate data analyses were done to obtain frequencies and proportions, and to determine the relationship between malaria parasitaemia and associated factors, respectively. The variables that were positive in the bivariate models were the only ones included in the multivariate model. Confidence level of 95% was used for level of significance.

### 2.6 Ethical clearance and consent to participate

This study was approved by the Ethical Research Committee of the Nasarawa State Ministry of Health [Ref: S/MOH/843/2014]. A written informed consent was obtained from all pregnant women prior to their enrolment in the study. Confidentiality of the participants and the information provided were assured and maintained throughout the study period.

## 3 Results

Overall, 242 pregnant women participated in the study. Their mean age (± SD) was 25.5 ± 5.5 years. Seventy women (28.9%) were recruited in the first trimester of pregnancy (≤ 13 gestational weeks). Those with asymptomatic malaria parasitaemia were 55, giving a prevalence of 22.7% (95% CI: 18.0–28.7 %). Among the study participants, 76 (31.4%) had no formal education, while 83 (34.3%) had at most a primary level of education. In total, 166 (68.6%) of the study participants had formal education and they were mostly civil servants. 153 Women (63.2%) were multigravidae (Table 1).

**Table 1:** Socio-demographic characteristics of women participating in the study (n =242).

Characteristics	n	%
*Age (years)*		
15 – 24	104	42.9
25 – 34	118	48.8
≥ 35 years	20	8.3
*Occupation*		
Civil servant	158	65.3
House wife	19	7.9
Business	53	21.9
Student	12	5.0
*Educational level*		
None	76	31.4
Primary	83	34.3
Secondary	65	26.9
Tertiary	18	7.4
*Gravidity*		
Primigravidae	89	36.8
Multigravidae	153	63.2

Factors significantly associated with asymptomatic malaria parasitaemia were Age < 25years [OR: 3.0, CI: 1.6 -5.6], low haemoglobin level (anaemia) [OR: 2.0, CI: 1.13.8], non regular use of LLIN [OR: 2.9, CI: 1.4-5.8], and being a primigravid [OR: 2.1, CI: 1.2-3.9] (Table 2). Using the multivariate logistic regression model, independent determinants of asymptomatic malaria parasitaemia were age < 25years [AOR: 2.4, CI: 1.2-4.9] and non-regular use of LLIN [AOR: 2.4, CI: 1.1-5.1] (Table 3).

**Table 2:** Factors associated with asymptomatic *Plasmodium falciparum* among the study participants. OR=Odds ratio; CI= Confidence interval.

Variable	Parasite positive n (%)	Parasite negative n (%)	Total n (%)	OR	95% CI
*Age (years)*					
< 25 years	35 (33.7)	69 (66.3)	104 (43.0)	3.0	1.6 - 5.6*
≥ 25 years	20 (14.5)	118 (85.5)	138 (57.0)		
*Haemoglobin*					
Anaemic [<11.0g/dl]	36 (28.6)	90 (71.4)	126 (52.1)	2.0	1.1 - 3.8*
Not Anaemic [≥11.0g/dl]	19 (16.4)	97 (83.6)	116 (59.1)		
*Regular LLIN use*					
No	17 (40.5)	25 (59.5)	42 (17.4)	2.9	1.4 - 5.9*
Yes	38 (19.0)	162 (81.0)	200 (82.6)		
*Gestation*					
First Trimester	10 (14.3)	60 (85.7)	70 (28.9)	0.5	0.2 - 1.0
Other Trimester	45 (26.2)	127 (73.8)	172 (71.1)		
*Gravidity*					
Primigravidae	28 (31.5)	61 (68.5)	89 (36.8)	2.1	1.2 - 3.9*
Multigravidae	27 (17.6)	126 (82.4)	153 (63.2)		
*Education*					
≤ Primary	38 (23.8)	122 (76.3)	160 (66.1)	1.2	0.6 - 2.3
≥ Secondary	17 (20.7)	65 (79.3)	82 (33.9)		
*Occupation*					
Unemployed	43 (25.3)	127 (74.7)	170 (70.2)	1.7	0.8-3.4
Employed	12 (16.7)	60 (83.3)	72 (29.8)		

* P<0.05

**Table 3:** Multivariate analysis of factors associated with asymptomatic *Plasmodium falciparum* among the study participants (n=242). AOR=Adjusted odds ratio; CI=Confidence interval.

Factor	AOR	95% CI
*Age (years)*		
< 25	2.4	1.2-4.9*
≥ 25	1	
*Regular LLIN use*		
No	2.4	1.1-5.1*
Yes	1	
*Haemoglobin*		
Anaemic [<11.0g/dl]	1.8	0.9-3.4
Not Anaemic [≥11.0g/dl]	1	
*Gravidity*		
Primigravidae	1.3	0.7-2.6
Multigravidae	1	

*P<0.05

## 4 Discussion

The results from this study showed a prevalence of 22.7% for asymptomatic malaria parasitaemia using microscopy among pregnant women attending antenatal clinic in Nasarawa-Eggon area. Women in the younger age group (< 25 years) were mostly infected and the non-regular use of an LLIN was an independent risk factor.

This prevalence is consistent with results of a study of pregnant women attending ANC clinic in Lagos, south-west Nigeria but lower than that of a similar study from a tertiary hospital in Abuja, north central Nigeria [21]. Studies from other developing countries with similar malaria endemicity and transmission intensity such as Cameroon, Democratic Republic of Congo (DRC), South Ethiopia, and Bangladesh, reported lower prevalence of between 2-21% using the same methods [9,22-24]. The varying prevalence could be due to the tropical savannah climate in most parts of Nigeria and the long duration of the rainy season (May to October) in Nassarawa-Eggon area.

The main species of malaria parasite identified in this study was *Plasmodium falciparum*. This is consistent with findings of studies done in Lagos, southwest Nigeria on factors associated with risk of malaria infection among pregnant women, and in Abuja, north central Nigeria on the prevalence of malaria parasitaemia among asymptomatic pregnant women [8,14]. This is not surprising since *P. falciparum* has been reported to account for about 94.8% of all malaria cases in Nigeria [25].

This study found age <25 years as a significant predictor of asymptomatic malaria parasitaemia. The results of multivariate analyses showed that the odds of developing asymptomatic malaria parasitaemia in women <25 years was 2.4 times the odds in women 25 years and older. This corroborates the fact that primigravidae have a higher risk of developing asymptomatic malaria parasitaemia and severe illness compared to multigravidae; which is attributed to a decrease in cell-mediated and humoral immunity in the first pregnancy [6,8]. This result is consistent with findings reported in Abuja, Nigeria and Libreville, Gabon, where primigravidae and young pregnant women were found to be more susceptible to malaria infection [8,26]. This finding is also consistent with a study done in Lagos, Nigeria on the risk of malaria infection in pregnant women in which maternal age less than 20 years old was found to be a significant risk factor for malaria parasitaemia during pregnancy [14].

Although being primigravid was significantly associated with asymptomatic malaria parasitaemia using bivariate analysis, it did not remain significant after controlling for age, haemoglobin level, and use of an LLIN. Age and gravidity are usually highly correlated unlike the findings of our study [9]. This is surprising because this study found the younger aged group to be the most infected. It could therefore be the effect of confounding from the higher proportion of the study group who are the multigravidae and the older aged group.

The use of an LLIN, which is one of the key preventive strategies of malaria in pregnancy, has been reported to substantially reduce the risk of malaria during pregnancy [27]. The use of LLINs as a preventive strategy for malaria during pregnancy as found in this study corroborates findings of a study in Africa and Thailand in which a systematic review of randomised trials on protection against malaria with LLINs was carried out [27]. Our finding that not using an LLIN increases the risk of malaria parasitaemia is also consistent with studies done in Anambra state, south-east Nigeria, where the use of LLIN was found to reduce the number of infective mosquito bites in a variety of ecologic settings [28]. This therefore indicates the necessity for creating awareness on the effective use of LLINs amongst pregnant women and the general population.

We found a statistically significant association between anaemia [< 11.0g/dl] and parasitaemia using bivariate analysis, although the association was not significant using multivariate analysis. The finding is similar to that of a study done in Calabar, Nigeria which reported lack of statistical significance on the prevalence of anaemia among primigravid and multigravid pregnant women with asymptomatic *P. falciparum* infection. However, there is still the need to raise emphasis on the importance of malaria in pregnancy and its implication on the mother and the fetus if not identified and treated. *Plasmodium falciparum* infection has been reported to result in the destruction of both young and new red blood cells which can lead to anaemia [26].

This study is limited because it was hospital based and not community based; hence it may not be generalised, but the populations are still similar. Another limitation was not being able to use Polymerase Chain Reaction (PCR) to rule out symptomatic malaria parasitaemia due to limited resources. However, the study has provided evidence-based information for policy direction in the study area and across Nigeria to intensify efforts at malaria prevention and focus interventions especially among the younger pregnant women. It has also provided evidence for the effectiveness of LLINs and the importance of appropriate use thereof.

## 5 Conclusions

This study provides evidence that a significant number of pregnant women with malaria parasitaemia but who are asymptomatic are found in Nassarawa-Eggon LGA. Younger women who also tend to be primigravidae, and those with low haemoglobin levels, are more at risk. Therefore malaria prevention interventions should be targeted at these groups especially with the use of LLINs, which has been shown to be effective in preventing malaria.
